# Rapid multistep kinetic model generation from transient flow data†
†Electronic supplementary information (ESI) available. See DOI: 10.1039/c6re00109b



**DOI:** 10.1039/c6re00109b

**Published:** 2016-10-03

**Authors:** Christopher A. Hone, Nicholas Holmes, Geoffrey R. Akien, Richard A. Bourne, Frans L. Muller

**Affiliations:** a Institute of Process Research and Development , School of Chemistry and School of Chemical and Process Engineering , University of Leeds , LS2 9JT , UK . Email: R.A.Bourne@leeds.ac.uk ; Email: F.L.Muller@leeds.ac.uk; b Department of Chemistry , Lancaster University , Lancaster , LA1 4YB , UK

## Abstract

Today, the generation of kinetic models is still seen as a resource intensive and specialised activity. We report an efficient method of generating reaction profiles from transient flows using a state-of-the-art continuous-flow platform. Experimental data for multistep aromatic nucleophilic substitution reactions are collected from an automated linear gradient flow ramp with online HPLC at the reactor outlet. Using this approach, we generated 16 profiles, at 3 different inlet concentrations and 4 temperatures, in less than 3 hours run time. The kinetic parameters, 4 rate constants and 4 activation energies were fitted with less than 4% uncertainty. We derived an expression for the error in the observed rate constants due to dispersion and showed that such error is 5% or lower. The large range of operational conditions prevented the need to isolate individual reaction steps. Our approach enables early identification of the sensitivity of product quality to parameter changes and early use of unit operation models to identify optimal process-equipment combinations *in silico*, greatly reducing scale up risks.

## Abstract 2

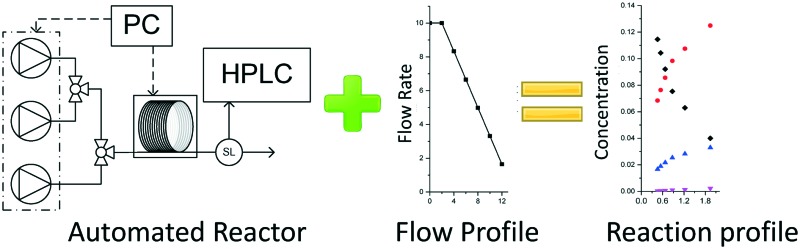
S_N_Ar reaction profiles were generated using an automated reactor, collected in less than 3 hours, and allowed accurate estimation of kinetic parameters.

Over the past decade flow technologies have become established for the discovery and manufacture of active pharmaceutical ingredients.^1,2^ A plethora of synthetic organic methods have been developed using flow reactors.^3,4^ However the development of new strategies that give fundamental understanding of complex reaction systems using flow reactors has not received as much attention.^5,6^ In this communication, we demonstrate the rapid collection of experimental data using an automated flow reactor for the generation of kinetic models.

Reaction profiling for reaction kinetics is easily achieved under batch conditions due to the ability to collect multiple time points within a single experiment, often with only milligrams of material used.^7^ Flow reactors give precise control over the reaction parameters. The key benefit of flow is that the system reaches steady-state thus providing a consistent output quality. There are many examples of kinetic investigations in small-scale flow systems.^8^ For instance Reizman *et al.* used steady state flow measurements to fit four rate constants and four activation energies from a small number of flow profiles.^9^ The authors concluded that high confidence parameter estimates require isolation of the individual pathways by measurements starting from each intermediate.

Kinetic profiling in flow systems suffers from three significant issues: (i) significant time is required to get to steady state; (ii) flow experiments require unnecessary material usage, approx. 1.5 reactor volumes per measurement, due to the transient period prior to reaching steady-state,^10^ and (iii) dispersion in flow systems can influence the outlet concentration resulting in errors in the derived rate constants. In this paper we address these three issues by use of online measurements in a system with transient flow.

Several authors have generated kinetic data based on transient flows. Mozharov *et al.* applied a step change in flow rate to study a Knoevenagel condensation reaction using inline non-invasive Raman spectroscopy.^11^ The response analysis was hindered by convolution of the step change by ‘real system’ response times, and the approach is infeasible for analysis techniques with longer acquisition times, such as HPLC and GC. Moore *et al.* studied a Paal–Knorr pyrrole synthesis using an exponential flow ramp with online infrared spectroscopy.^12^ The effect of residence time and temperature were investigated, at constant inlet concentrations. The experimental data were successfully fitted to *a priori* rate expressions. Schaber *et al.* extended the approach in terms of model discrimination.^13^

This communication demonstrates a fully automated approach for collecting experimental data for kinetic model generation using transient flow data. The approach is illustrated using a nucleophilic aromatic substitution (S_N_Ar) of 2,4-difluoronitrobenzene **1** with pyrrolidine **2** in ethanol to give a mixture: desired product *ortho*-substituted **3**, *para*-substituted **4** and *bis*-adduct **5** as side products (Scheme 1).

**Scheme 1:**
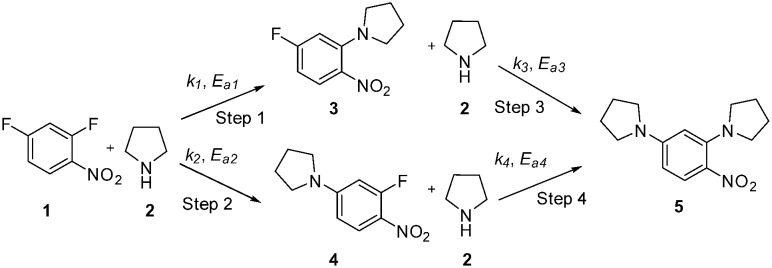
S_N_Ar reaction of 2,4-difluoronitrobenzene **1** with pyrrolidine **2**.

In this study an automated mesoscale flow reactor system (5 mL reactor volume, 0.79 mm internal diameter, 1.58 mm outer diameter) with online HPLC, see Fig. 1, was used. A linear gradient flow ramp, see Fig. 2, which allows for investigating a complete reaction profile from a single transient experiment was developed. A transient profile measurement starts by setting the maximum flow for all pumps. The flow rate ratio of P3 (pyrrolidine in EtOH) and P2 (EtOH) is varied at the beginning of each ramp to obtain different molar equivalents (1.5, 4 and 7) of pyrrolidine **2**. As a sudden increase in flow rate produces unstable flow, pump flow rates were kept constant long enough to establish steady-state. Subsequently the flow rate, *Q*, is slowed at a constant rate, *α* (0.836 mL s^–2^), so as to increase the residence time (*τ*_res_) over time *t*, whilst maintaining constant inlet concentrations. Reactor effluent is injected into the HPLC at 2 min time intervals. The pump flow ramps, reactor temperature and sample loop injection were automatically controlled by a MATLAB based computer program. Rather than isolate the individual pathways we extended the range of reaction conditions so as to over- and under-react significantly. Application of the full operational range allowed by the equipment *e.g.* from the mildest (*e.g.* dilute, low temperature) to the harshest (*e.g.* concentrated, high reagent to substrate ratios, high temperature) results in higher concentrations of intermediates and by-products. This represents a richer dataset that increases the confidence in parameters of the fitted kinetic motifs.^14^ Hessel *et al.* introduced the concept of novel process windows (high temperature, high pressure and high concentration) for accessing conditions in flow which are not typically accessed in conventional practice for organic synthesis in batch.^15^ Thus linear gradient flow ramps (Fig. 2) were developed to explore from the mildest (**2** = 1.5 mol eq., = 0.5 min and *T* = 30 °C) to the harshest conditions (**2** = 7 mol eq., = 2 min and *T* = 120 °C). Residence time points less than 0.5 min were not collected as transient effects dominate.^16^ The approach focuses on using reaction conditions that maximise the confidence in the kinetic parameter estimates.

**Fig. 1:**
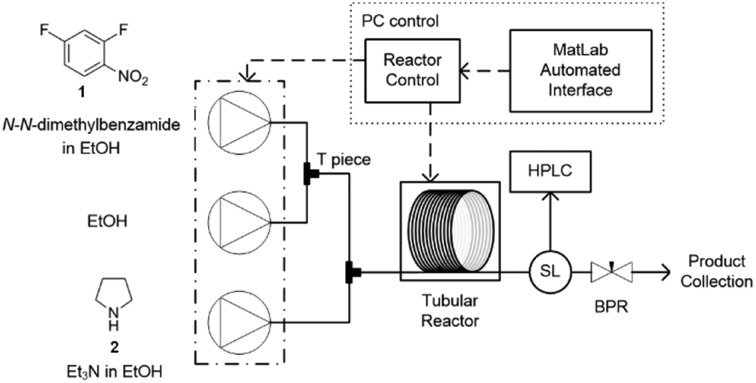
Automated continuous-flow reactor system equipped with software for controlled flow ramps. The reactor comprises of three Jasco PU-980 dual piston HPLC pumps which feed into Swagelok tee-pieces for mixing. A polar bear flow synthesiser (Cambridge Reactor Design, UK) was used for heating and cooling PTFE reactor tubing (5 mL internal volume, 0.79 mm internal diameter). A VICI Valco internal sample injector (SL) extracted aliquots of neat reaction for HPLC analysis. The composition of the reactor outlet was determined by online HPLC. Pressure control was achieved with a back-pressure regulator at the outlet of the system. The internal reactor temperature was measured by a thermocouple inserted into the centre of the reactor.

**Fig. 2:**
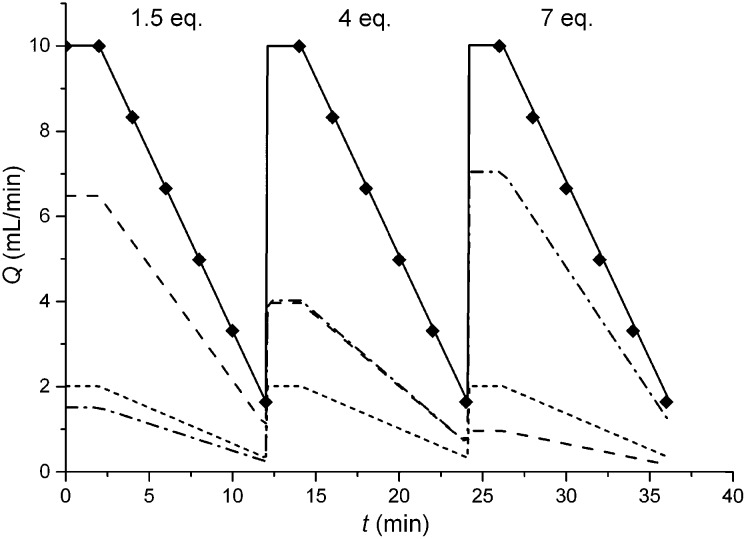
Changes in volumetric flow rate over time, where *Q*_P1_, *Q*_P2_, *Q*_P3_ and *Q*_total_ were for 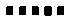 pump 1 (Ar in EtOH), 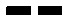 pump 2 (EtOH), 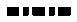 pump 3 (pyrrolidine in EtOH), 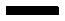 total volumetric flow rate, and ◆ HPLC injection respectively. The linear flow ramps correspond to pyrrolidine **2** to 2,4-difluoronitrobenzene **1** molar ratios: (i) 1.5 : 1, (ii) 4 : 1 and (iii) 7 : 1 using *Q*_total_ from 10 to 1.5 mL min^–1^.

The transformation of the data is shown in Fig. 3. For a linear flow ramp the velocity in the coil is described by:

1 *u*(*t*) = *β*(*T*)(*u*_o_ – *αt*)

where *u*_o_ is the initial velocity, *α* the rate of change and *β*(*T*) = 1 + *α*_v_(*T*_1_ – *T*_0_) is the correction for the thermal expansion of EtOH due to the temperature rise from ambient to the reactor temperature. A fluid element leaving the coil at time *t* will have travelled the length of the tube (*L*) and resided a total time *τ*_res_ so:2
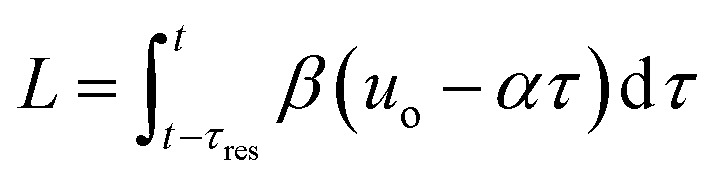



**Fig. 3:**
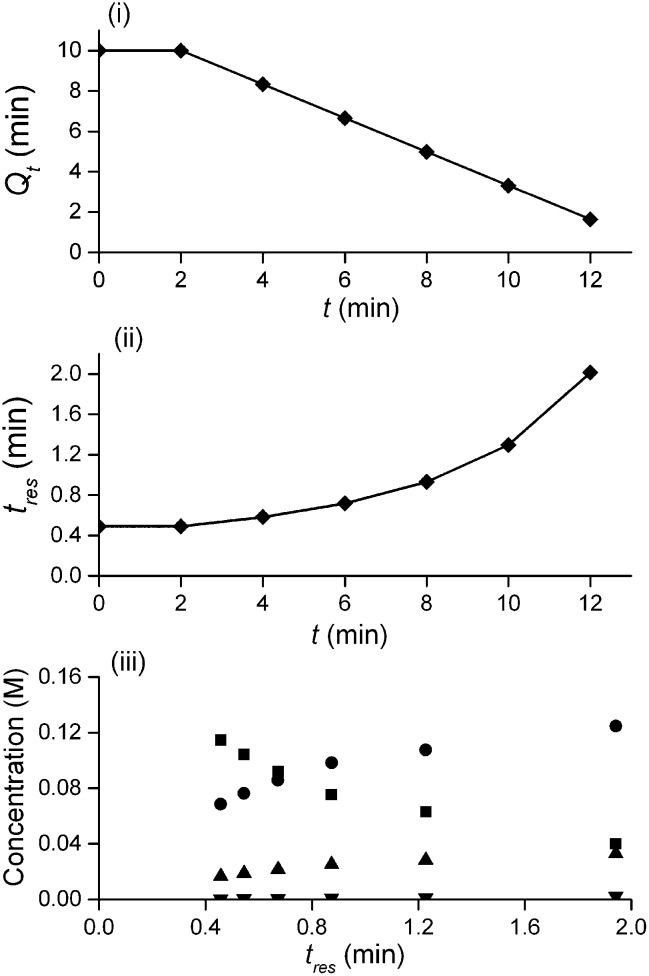
i) Total volumetric flow rate (*Q*_total_) change throughout the duration of a ramp, ♦ HPLC injection; (ii) residence time, *τ*, as a function of operation time, *t*; (iii) resulting concentration-residence time profile generated from a typical multi-step reaction scenario, ■ 2,4-DF **1**, *ortho*-**3**, *para*-**4**, bis-**5**.

Integration leads to a second order equation from which the residence time *τ*_res_ of a sample taken at time *t* can be worked out as:3
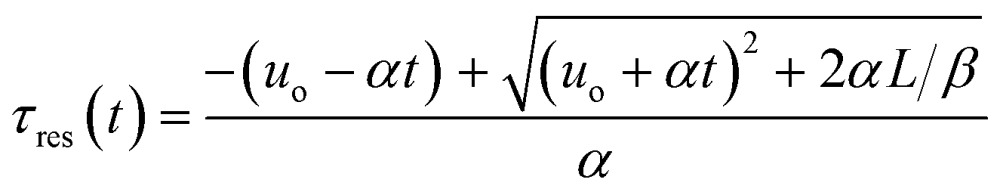



The method was applied to generate a series of concentration-time profiles at four temperature levels (30, 60, 90 and 120 °C) giving a total of 12 profiles (Fig. 5). The 72 data points shown in Fig. 5 were collected in less than 3 hours collection time. The reaction conditions were selected to give a wide range of conversions values for the different reaction components.

The data were then fitted to the kinetic scheme shown in Scheme 1, with different orders assessed with respect to the aromatic components and pyrrolidine (see ESI†). The kinetic motif in which all steps are second order gave the best fit. The rate constants were initially fitted at isothermal conditions (90 °C) using the Levenburg–Marquardt algorithm, a non-linear least squares algorithm, in DynoChem software (Scale-up Systems). Subsequently all the experimental data (72 data points) were simultaneously fitted to give all the kinetic parameters (Table 1). The kinetic model very closely corresponded to the experimental data, with *R* ^2^ = 0.9995 (Fig. 4). The rate constant *k*_1_ for *ortho*-**3** is 20 times larger than *k*_2_ of the *para*-**4** product formation and their activation energies are 33.3 ± 0.3 kJ mol^–1^ and 35.3 ± 0.5 kJ mol^–1^ respectively; thus temperature influences the rate, but not selectivity. Parameter uncertainties were all less than 4%. Even the rate parameters for the overreaction pathways identified with minimal uncertainty. Unsteady-state results were compared to results at steady-state, the two methods gave statistically similar results to data collected at steady-state conditions (ESI†). More aggressive conditions *e.g.* high molar equivalents of pyrrolidine and high temperature result in elevated formation of the bis adduct **5** product to which *k*_3_ and *k*_4_ were fitted with high confidence, thus preventing the need to synthesise and isolate each reaction component. Our approach offers significant time savings and minimises material consumption compared to a steady-state approach, addressing shortcomings (i) time required to reached steady-state for each measurement and (ii) material wastage reaching steady-state. Further material savings could be made using a flow system with a small internal volume, such as on a microliter scale as reported by McMullen *et al.* ^17^

**Table 1:** Kinetic parameter estimates and standard errors (SE) from the fitting based on 95% confidence level. Rate constants, *k*, are given at *T*_ref_ = 90 °C

	*k* ± SE (10^–2^ M^–1^ s^–1^)	*E*_a_ ± SE (kJ mol^–1^)
Step 1	57.9 ± 0.7	33.3 ± 0.3
Step 2	2.70 ± 0.06	35.3 ± 0.5
Step 3	0.865 ± 0.004	38.9 ± 1.5
Step 4	1.63 ± 0.11	44.8 ± 1.8

**Fig. 4:**
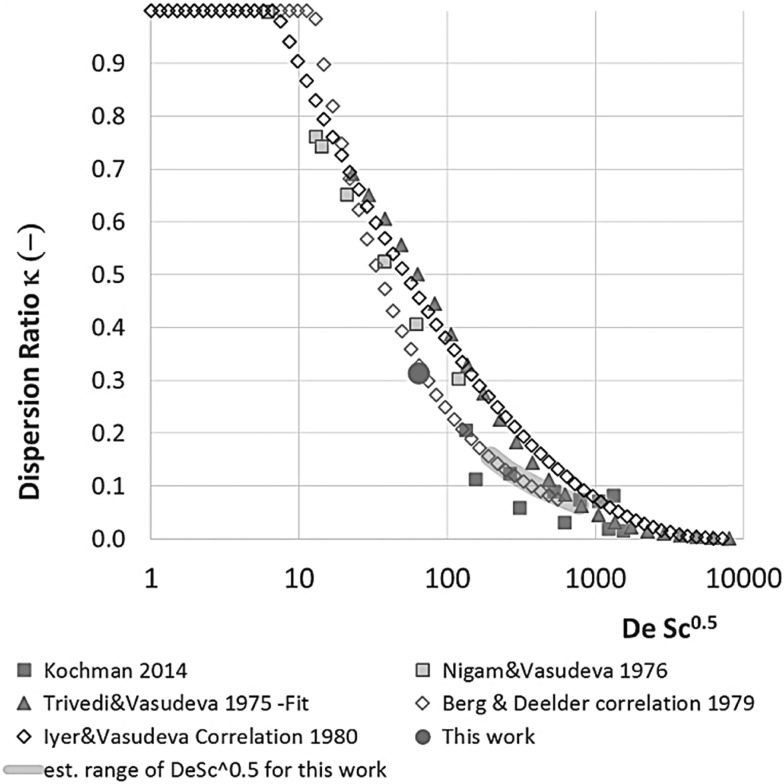
The dispersion ratio *κ vs.* DeSc^0.5^ for various literature data. Where possible data have or correlations have been taken from the papers. If the data could not be retrieved and no correlation was given the data has been fitted with a line that visually represents the data well.

Limitation (iii) concerned the influence of dispersion on the measured rate constants. If the coil reactor is described by a plug flow model the conversion for a first order reaction is:4
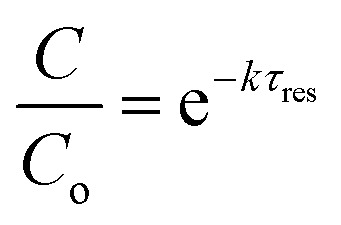



It is well established that dispersion can have a significant effect on the progress of a reaction.^18^ In the 1950s Taylor described dispersion in of a solute a straight pipe with laminar parabolic flow and this was modified by Aris,^19,20^ to give:5
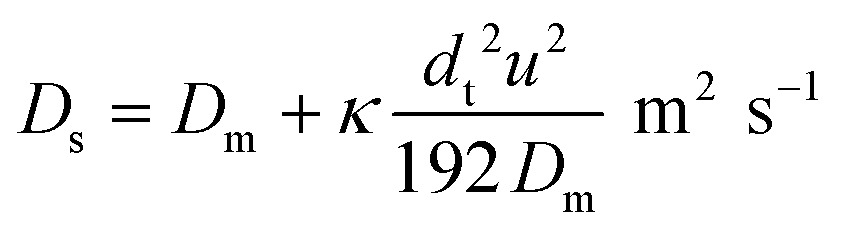



With *D*_S_ the dispersion coefficient, *D*_m_ the diffusion coefficient, *d*_t_ the tube diameter and *u* the mean velocity in the tube. In liquid systems the term *D*_m_ is negligible, and *κ* represents the ratio of dispersion in a conduit to dispersion in a straight tube with similar diameter; for a straight cylindrical tube under laminar flow *κ* = 1. The dispersion ratio *κ* is dependent on the channel geometry and the flow regime (*e.g.* laminar or turbulent). Laminar flow in coiled tubes deviates from the parabolic velocity profile as a result from centrifugal forces. So called Dean vortices form which introduce radial flow that reduces dispersion. The intensity of the radial flow is characterised by the Dean number:6
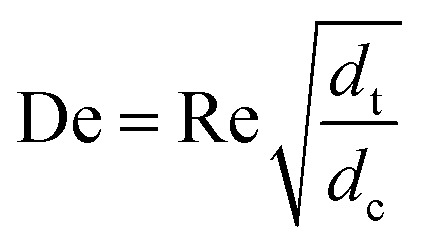



Re is the Reynolds number (*ρud*_t_/*μ*) and *d*_c_ the coil diameter (0.79 mm in our case). In the 1970s dispersion in coils under laminar conditions was studied experimentally in the group of Vasudeva in wide bore tube (4.4–20 mm),^21–23^ and by Van den Berg and Kockmann in 0.5 and 1 mm ID tubes.^24,25^ They found that the dispersion ratio *κ* reduces significantly below 1 (Fig. 4). Theoretical work by Janssen and later by Johnson showed dispersion ratio in coils may be correlated by DeSc^0.5^, where the Schmidt number (Sc) is defined as *μ*/*ρD*_m_.^26,27^ In the 1960s the effect of dispersion was coupled to a reaction system by Wehner^28^ to give a general equation for the effect of dispersion on the conversion of a solute due to a first order reaction with rate constant *k*:7
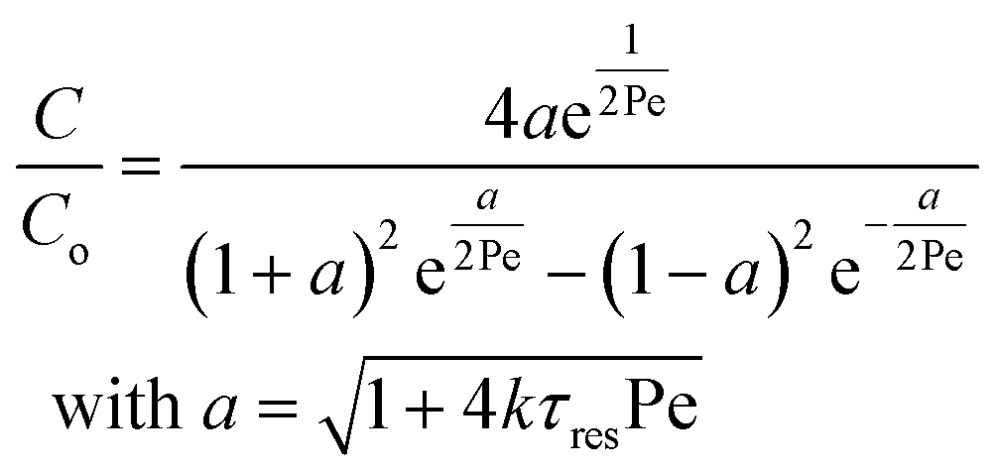



In which dispersion is characterised with the dimensionless Péclet number Pe = *D*/*uL*. Eqn (7) can be simplified for values of Pe < 0.05 by applying a second order Taylor series expansion for *a*:

8 *a* ≈ 1 + 2*kτ*_res_Pe – 2(*kτ*_res_Pe)^2^

where *kτ*_res_Pe = *kD*/*u* ^2^ is assumed to be small, therefore the dispersion is small and peaks remain symmetrical. Combining eqn (5) and (7), and the observation that (i) in liquid systems the term *D*_m_ becomes negligible compared to *D*_s_ and (ii) *τ*_res_ = *L*/*u*, it follows that:9
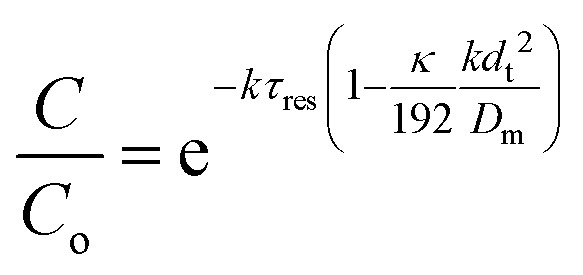



This shows that in a coil the observed rate constant *k*_obs_ may be given as:10
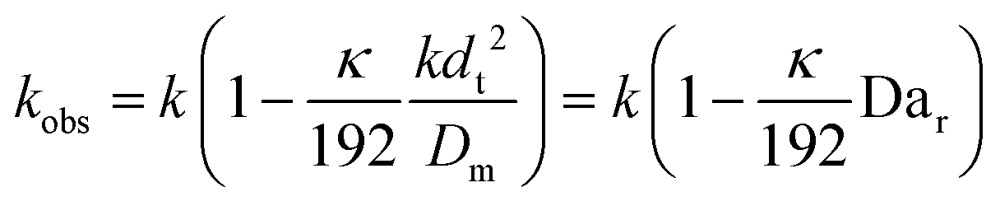
where the Damköhler number for radial diffusion may be defined as Da_r_ = *kd*_t_^2^/*D*_m_. The deviation *ε*_D_C__ in the observed rate constant *k*_obs_ obtained from the profiles measured in continuous flow in a coil with respect to the true rate constant *k* will be:11
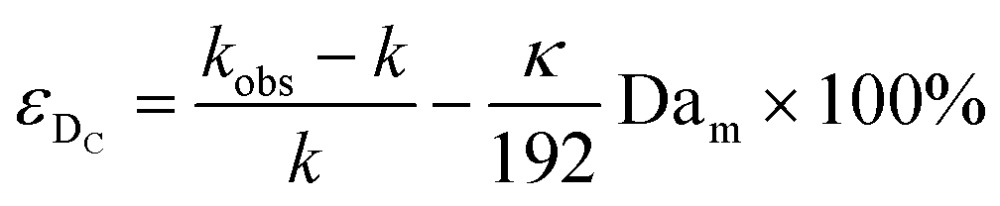



 Eqn (11) shows the kinetic constants will always be underestimated if dispersion is significant. The above result shows that for a tube with 1 mm internal diameter and a typical value for *D*_m_ = 0.8 × 10^–9^ m^2^ s^–1^ in EtOH (ref. 29) the reduction in rate constant is *ε*_D_C__ ≈ –500% *κk*. For instance, Durant measured the rate constant for the thermolysis of 1,3-dioxin-4-ones to be <0.015 s^–1^ in a 1 mm ID coil at DeSc^0.5^ = 13.8, 138 and 1380.^30^ This corresponds to a deviation *ε*_D_C__ ≤ –5.6%, –1.5%, 0.38% respectively (*κ* from Fig. 4), which corresponds well to their observation of a negligible effect of dispersion. In a 1 mm coil system with *κ* < 0.1 rates as fast as 0.1 s^–1^ can be measured accurately. Smaller tube diameters will reduce the error, as *κ*Da_r_ reduces.

To assess the impact of dispersion on the second order rate constants for conversion of 2,4-difluoronitrobenzene **1**, at a particular excess of pyrrolidine the first order rate constant *k* may be approximated by:

12 *k* ≈ (*k*_1_ + *k*_2_)*C*_2,0_

In our system we measured an *F* curve at 6 min residence time giving Da_r_ = 788 and DeSc^0.5^ = 65 (ESI†). This resulted in a dispersion ratio of *κ* = 0.31 which corresponds well with the data from Van den Berg.^24^ Using the correlation from Van den Berg for *κ* in coiled tubes we estimate that for the experimental conditions used to generate the data on which Table 1 is based *κ*, is in the range of 0.05 to 0.15 (Fig. 4). Using eqn (11) the extent to which *k*_1_ + *k*_2_ is underestimated can be evaluated for profiles where the starting material concentration deviates significantly from zero *e.g.* plots i, ii and v for **1** in Fig. 5 (for the profiles where the reactant has fully converted or no stating material is available at all residence times no sensible estimate dispersion effect on *k* can be made). To be conservative we assumed a value of *κ* = 0.15 for all errors in Table 2. The unshaded cells represent profiles with significant concentrations of reacting components. The fitted motif is thus fit for purpose with respect to evaluate parameter sensitivity, alternate unit operations and scale-up.

**Fig. 5:**
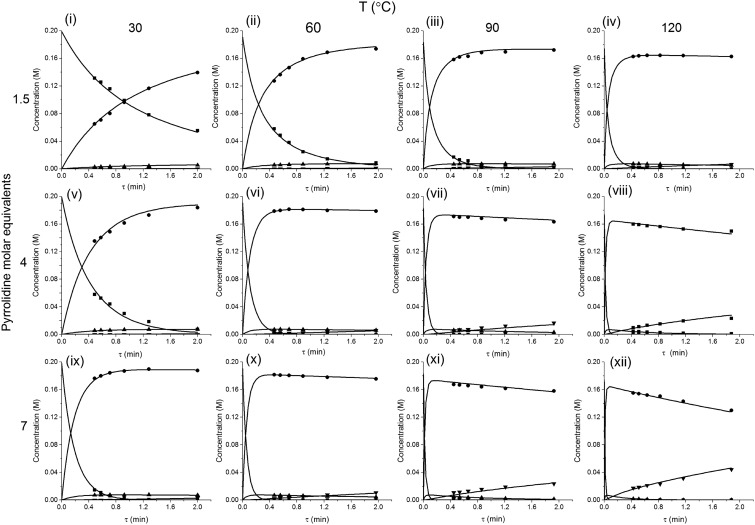
Concentration-time profiles from simultaneous parameter fitting, points = experiments ■ 2,4-DF **1**, *ortho*-**3**, *para*-**4**, bis-**5**, lines = model using Table 1 kinetic parameter estimates (i–xii).

**Table 2:** Estimated predicted % effect in rate constants *k*_1_ + *k*_2_ and *k*_3_ + *k*_4_ from dispersion. The cells highlighted in grey show where the deviation in concentration is too low to significantly affect the fitting of the rate constants

## Conclusions

An automated continuous-flow platform with quantifiable online analysis has been developed as an enabling tool for the rapid and economic collection of kinetic profiles. In less than 3 hours, 12 reaction profiles were collected; sufficient to fit a kinetic motif consisting of 4 reactions, 8 fitting parameters, with less than 4% uncertainty. Furthermore, it has been demonstrated that the effect of dispersion in these system results in an underestimation of the rate constants by 5% or less. The efficiency of the linear flow ramp is reduced if the product analysis takes a long time, however; with recent advances in analytical techniques, *e.g.* UPLC and FlowIR many reaction systems can be analysed successfully within seconds to minute timescales. Data collected from transient flow profiles gave statistically similar results to data collected at steady-state further validating the flow ramp approach. The combination of a linear gradient flow ramp and extreme conditions far away from the preferred operating point, easily accessed in continuous-flow reactors, enables rapid data generation with a quality suitable for fitting parameters of multistep kinetic motifs. Our approach allows kinetic models to be generated much earlier in process development, allowing early estimation of the sensitivity of product quality to input parameter changes. The model can be applied *in silico* to simulate alternative optimisation scenarios, equipment configurations, and to achieve significant reductions in scale up risks and costs.
