# Supplementary material for: Rapid multistep kinetic model generation from transient flow data
Source: React Chem Eng. 2016 Oct 3;2(2):103–8. doi: 10.1039/c6re00109b (PMC5436494; doi:10.1039/c6re00109b)
Supplement: Supplementary file 1 [file RE-002-C6RE00109B-s001.pdf]

Electronic Supplementary Material (ESI) for Reaction Chemistry & Engineering.
This journal is © The Royal Society of Chemistry 2016

Electronic Supporting Information (ESI)

Contents

S1 Experimental details 2

S2 HPLC analysis 3

S3 Model determination 4

S4 Joint confidence intervals 4

S5 Parameter correlation 5

S6 Exploring a wide design space 6

S7 Kinetic parameters for morpholine S_NAr system 7

S8 Experimental data and model fit to morpholine S_NAr system 8

S9 Linear gradient flow ramps for 2,4-fluoronitrobenzene 10

S10 Comparison to measurements under steady-state conditions 11

S11 Influence of dispersion on kinetic parameter estimation 12

S12 Dispersion experiment 16

S13 References 17

S1 Experimental details

A schematic of the reactor configuration can be seen in Figure 1 and a labelled photograph is shown in Figure S1. Three Jasco PU-980 dual piston pumps were connected separately to solutions of (A) 1 M 2,4-difluoronitrobenzene **1** and internal standard *N*,*N*-dimethylbenzamide (0.25 equiv.) in EtOH, (B) 2 M pyrrolidine **2** or morpholine **3** and triethylamine in EtOH, and (C) neat EtOH. The molar ratio of triethylamine to morpholine (1.1:1) was kept constant throughout the study. The concentration of the reagents within the reactor was determined by the relative pump flow rates based on the automated linear gradient flow ramps. The automated linear gradient flow ramps were operated at four different reaction temperatures for each reaction example. MATLAB was used to send the temperature to reactor, control the pump flow rates and to trigger the sample loop to inject into the HPLC. Manipulation of the pump flow rates allowed for a range of residence times and reactant concentrations to be investigated. PTFE tubing (Polyflon, Stafford, UK) was used with 0.031 in. internal diameter and 0.063 in. outer diameter. The flow streams were mixed *via* two Swagelok tee-pieces. A Polar Bear Flow Synthesiser (Cambridge Reactor Design, UK) was used for heating and cooling the PTFE reactor tubing which had an internal volume of 5 mL. A VICI Valco internal sample injector (0.06 μL volume) extracted aliquots of neat reaction for HPLC analysis without prior quench or dilution. A 10 bar backpressure regulator was installed downstream of the sample injector. The high heat transfer provided consistent temperature across the tubing; this assumption was tested through repeat experimentation of a linear flow ramp gradient with inclusion of tubing (each 3 mL volume) for the pre-heating of the three inlet streams prior to mixing.

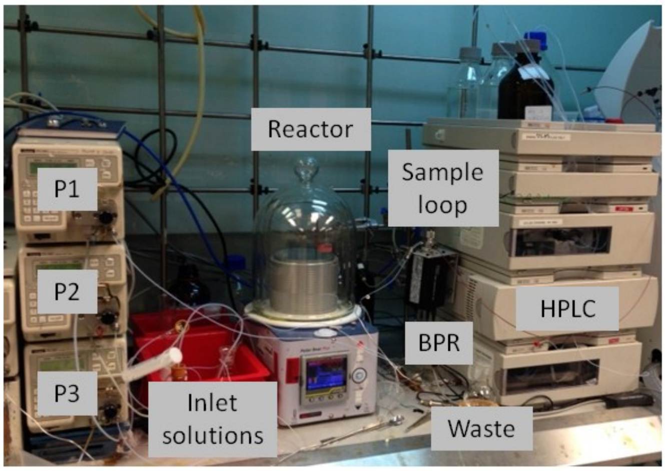

Figure S1. *Labelled image for the automated continuous-flow reactor platform.*

S2 HPLC analysis

HPLC quantitative analysis was performed on an Agilent 1100 series LC using an Ascentis Express C18 reverse phase column (5 cm length, internal diameter 4.6 mm and 2.7 µm particle size), a water/acetonitrile mobile phase, and a 254 nm wavelength detector. A VICI Valco internal sample injector (0.06 µL volume) extracted aliquots of neat reaction for HPLC analysis without prior quench or dilution. All reported values were nonisolated and attained by HPLC based on normalisation of response factors using *N*-*N*-dimethylbenzamide as an internal standard. The HPLC was calibrated for the different reaction components. A typical HPLC is shown in Figure S2. For the pyrrolidine **2** reaction system the compounds were separated using an isocratic (51% water/49% acetonitrile), 1.5 mL min^{-1} total flow rate and a 2 min run time. For the morpholine **6** reaction system the compounds were separated using an isocratic (60% water/40% acetonitrile), 1.5 mL min^{-1} total flow rate and a 1.66 min run time.

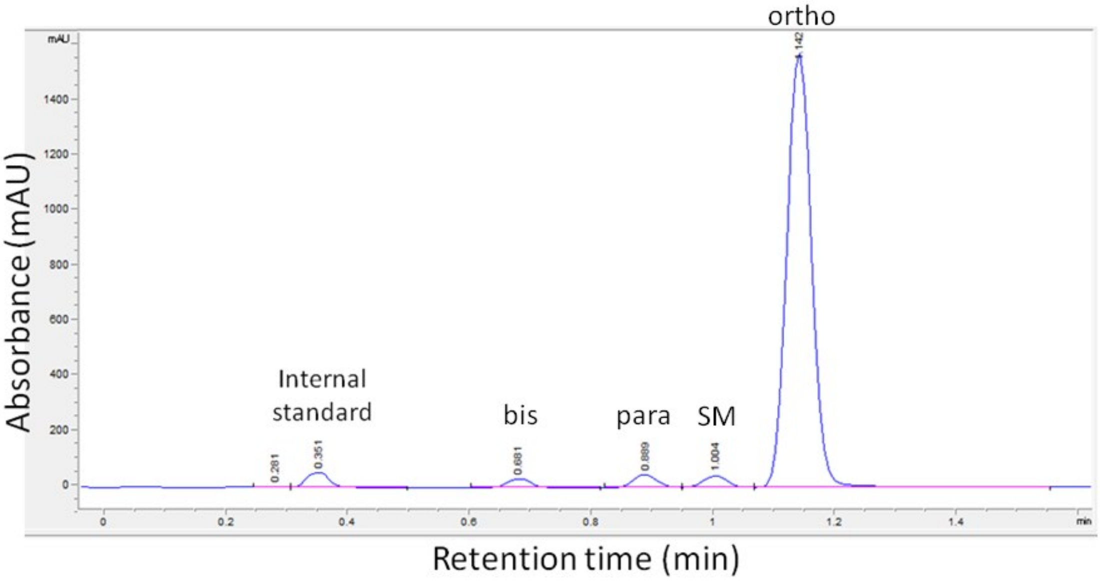

Figure S2. *Example HPLC chromatogram.*

S3 Model determination

The experimental data was used to compare five different proposed kinetic models, with different concentration dependencies with respect to the aromatic component and pyrrolidine for each step in Scheme 1.

$$\text{Reaction step rate} = k_x[\text{aromatic}]^y[\text{pyrrolidine}]^z$$

Based on different statistical analysis techniques, the second order model $M_{1,1}$ gave a significantly better fit (Table S1), which is in line with expectations based on initial kinetic investigations by Bunnett. The model selection criteria (MSC) is an analysis technique used to compare different models with the model with the higher MSC being the better model.

Table S1. Comparison of model fit for different reaction orders for the reaction steps.

Statistics	$M_{1,0}$	$M_{0,1}$	$M_{1,1}$	$M_{2,1}$	$M_{1,2}$
Sum of squares	11.1	11.1	1.85	10.7	12.1
Variance	0.0396	0.0397	0.00660	0.0383	0.0434
Standard deviation	0.199	0.200	0.0812	0.196	0.208
Model selection criteria	4.78	4.80	7.50	3.50	4.20

S4 Joint confidence intervals

The covariance matrix measures how much two random variables change together, and was used to estimate the joint confidence intervals. The parameter joint confidence regions were examined to observe the multi-parameter dependence of one parameter upon another, a change in one parameter value will cause a change in the other parameter value (Figure S3). An elongated elliptical confidence region shows that the rate constant and activation energy for each pathway are correlated. A reduction in size of the joint confidence regions is achieved through exploration of sensitive regions of the experimental space. There will always be some correlation between the activation energy and rate constant for a particular step. The joint uncertainty was minimised for each parameter through conducting sufficient experiments where change would be observed; the uncertainties for k_3 and E_{a3} and for k_4 and

E_{a4} were minimised by using a high molar excess of pyrrolidine **2** and operating the reactor at high temperature.

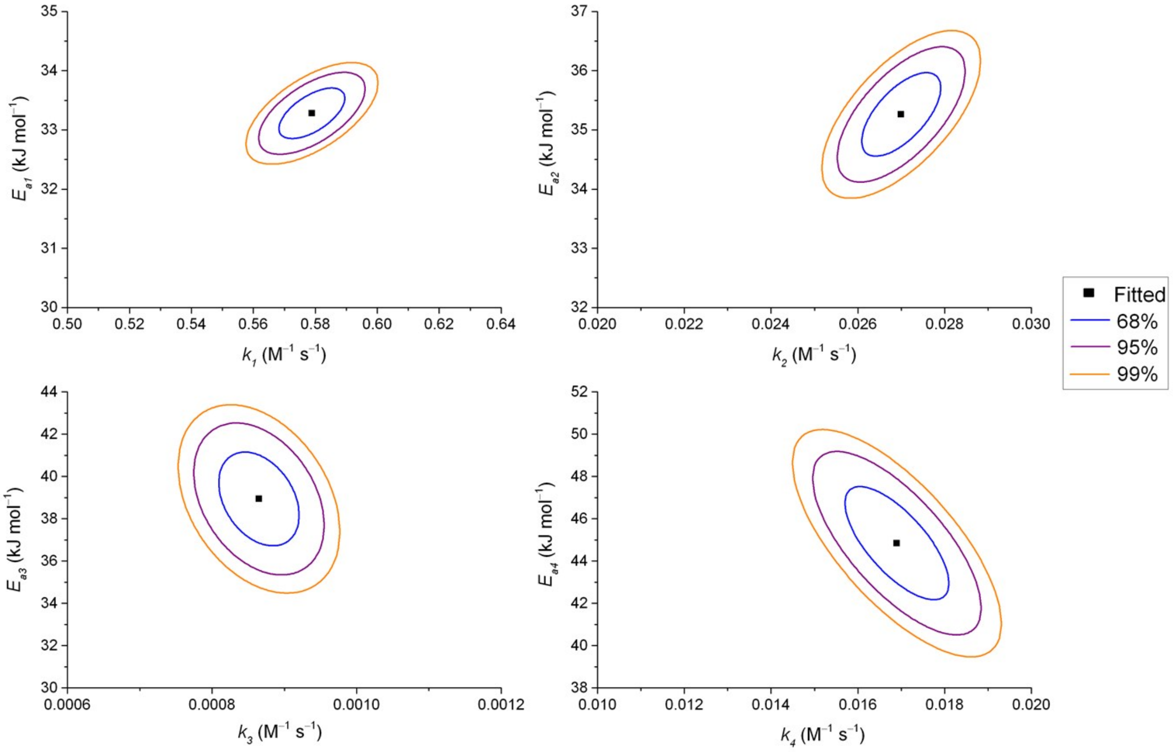

Figure S3. *68, 95 and 99% joint confidence regions for the estimated parameters after all experiments for pyrrolidine **2** system.*

S5 Parameter correlation

The correlation matrix is a normalised form of the covariance matrix, and describes the strength and direction of the relationship between two parameters. The correlation matrix is generated as an output as part of the DynoChem fitting report (Figure S4). A correlation value near zero means one parameter can be changed without expecting a change in another parameter; −1 negatively correlated, +1 positively correlated. The highest correlation observed was between each rate constant and its activation energy as shown by the joint confidence intervals and the correlation matrix. The correlation matrix clearly shows that a sufficient experimental design space had been explored to minimise the correlation between different parameters.

$$\rho(V_\theta) = \begin{array}{c|cccccccc|} & k_1 & k_2 & k_3 & k_4 & E_{a1} & E_{a2} & E_{a3} & E_{a4} \\ \hline k_1 & 1.000 & 0.450 & 0.060 & -0.070 & 0.600 & 0.310 & -0.010 & 0.030 \\ k_2 & 0.450 & 1.000 & -0.460 & 0.540 & 0.290 & 0.640 & 0.130 & -0.220 \\ k_3 & 0.060 & -0.460 & 1.000 & -0.690 & 0.010 & -0.190 & -0.350 & 0.530 \\ k_4 & -0.070 & 0.540 & -0.690 & 1.000 & 0.009 & 0.200 & 0.500 & -0.740 \\ E_{a1} & 0.600 & 0.290 & 0.010 & 0.009 & 1.000 & 0.450 & 0.040 & -0.500 \\ E_{a2} & 0.310 & 0.640 & -0.190 & 0.200 & 0.450 & 1.000 & -0.140 & 0.120 \\ E_{a3} & -0.010 & 0.130 & -0.350 & 0.500 & 0.040 & -0.140 & 1.000 & -0.640 \\ E_{a4} & 0.030 & -0.220 & 0.530 & -0.740 & -0.500 & 0.120 & -0.640 & 1.000 \end{array}$$

Figure S4. *Correlation matrix for the S_NAr pyrrolidine reaction kinetic parameters.*

S6 Exploring a wide design space

The dimensionless time plot, see Figure S5, represents the relative reactivity of all the input conditions used in the experimental study, a higher number indicates higher reactivity. The plot shows that experimental data were collected from very mild to aggressive conditions, within the constraints of the equipment, were studied thus allowing the kinetics for the whole reaction scheme to be fitted. A parity plot, a plot of the fitted responses vs. the observed responses to assess the quality of the fit is shown in Figure S6.

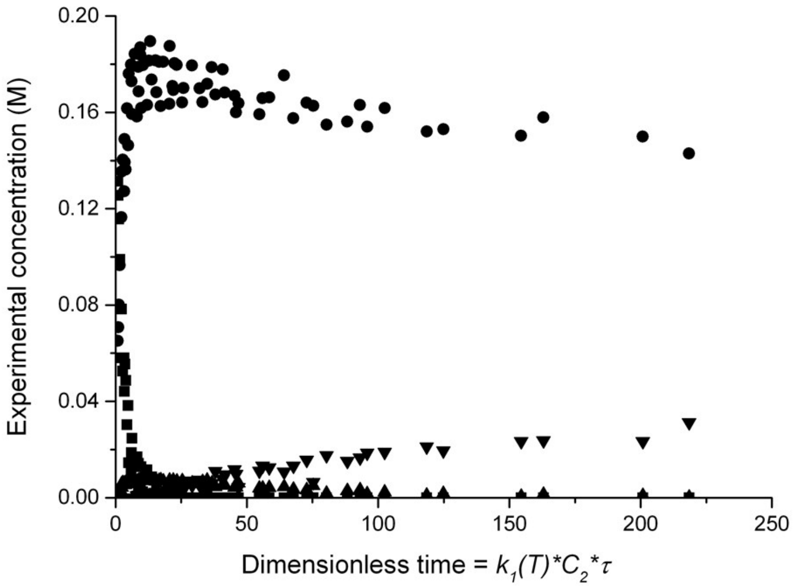

Figure S5. Dimensionless time plot, points = experiments ■ 2,4-DF **1**, ● *ortho*-**3**, ▲ *para*-**4**, ▼ *bis*-**5**.

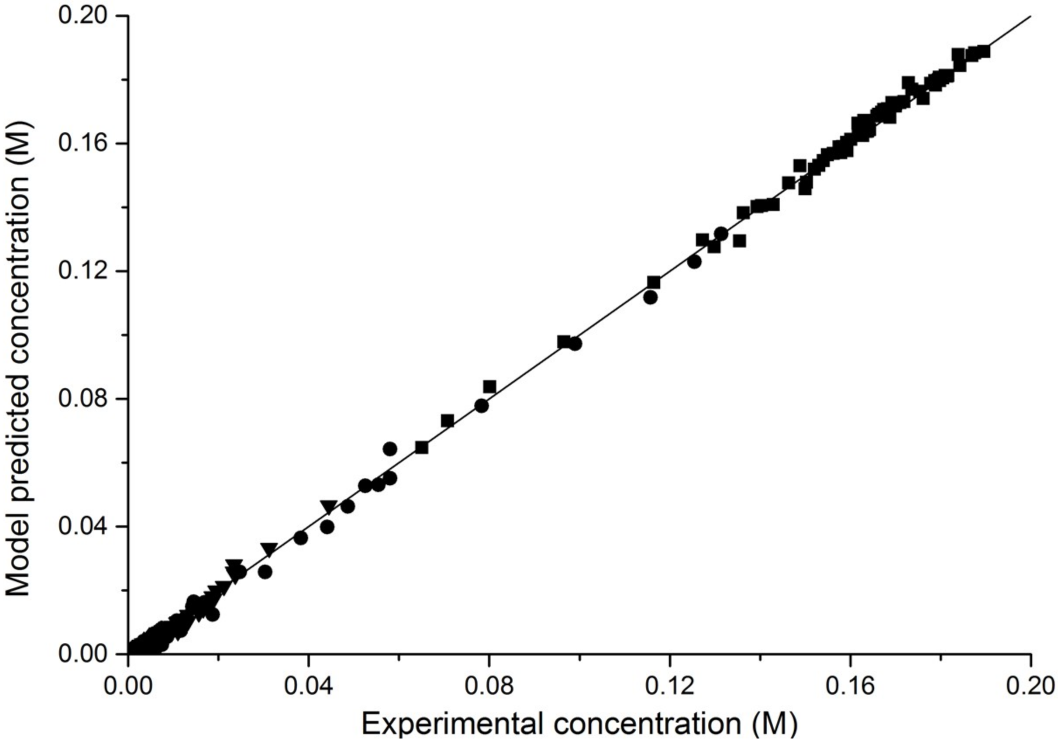

Figure S6. Parity plot to show excellent correspondence between experimental and model predicted concentration. Points = experiments ■ 2,4-DF 1, ● *ortho*-3, ● *para*-4, ▼ *bis*-5, line y = x.

S7 Kinetic parameters for morpholine S_NAr system

Scheme S1. *S_NAr reaction of 2,4-difluoronitrobenzene* ***1*** *with morpholine* ***6****.*

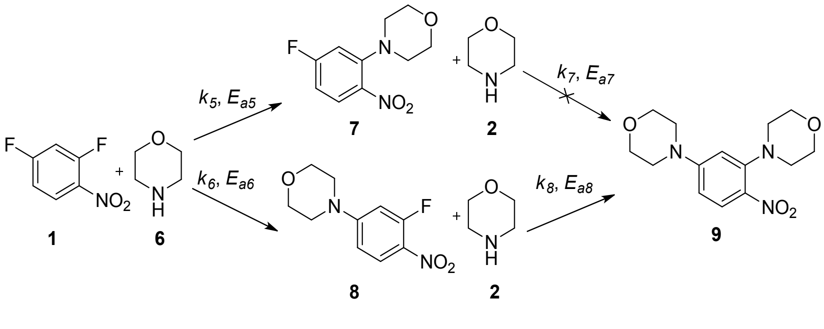

Table S2. *Kinetic parameter estimates and standard errors based on 95% confidence level. Rate constants, k, are given at T_{ref} = 90 °C.*

	k ± SE (10^{-3} M^{-1} s^{-1})	E_a ± SE (kJ mol^{-1})
Step 1	23.70 ± 0.04	38.2 ± 0.3
Step 2	6.90 ± 0.01	32.9 ± 0.5
Step 3	Converged to zero	Converged to zero
Step 4	1.500 ± 0.005	40.8 ± 1.5

S8 Experimental data and model fit to morpholine S_NAr system

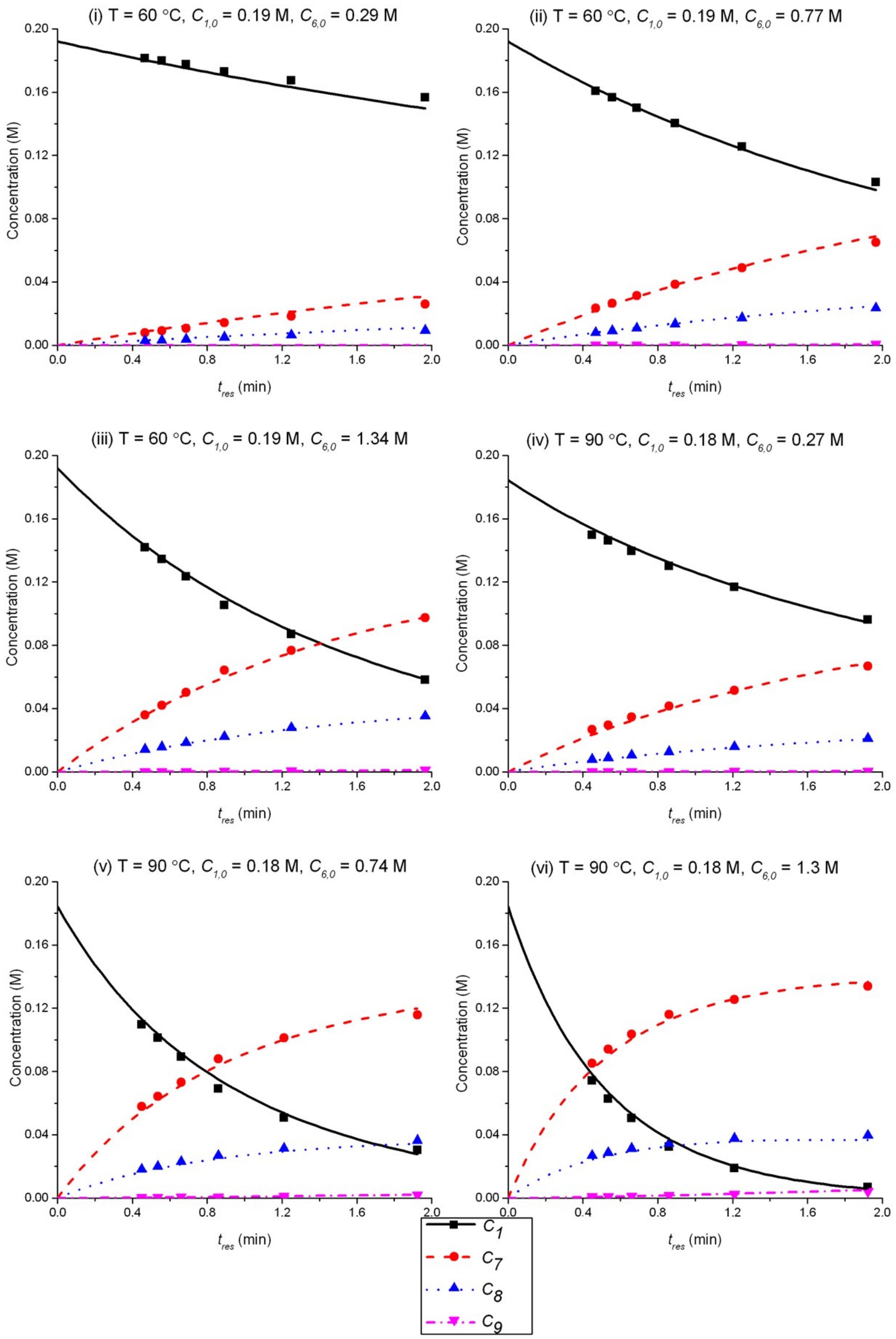

Figure S7. *Concentration-time profiles for morpholine reaction system from simultaneous parameter fitting using Table S1 kinetic parameter estimates, points = experiments, lines = model.*

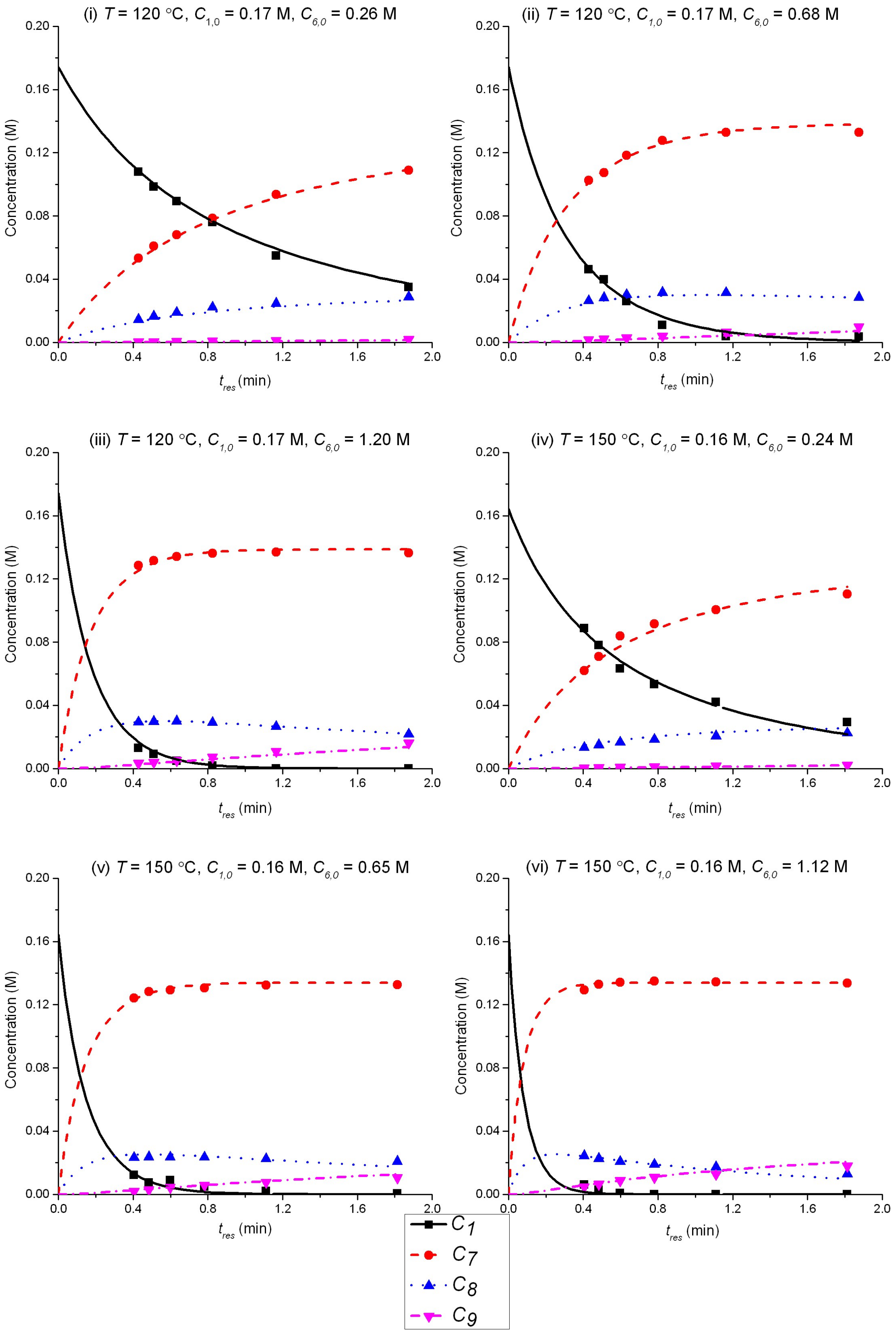

Figure S8. *Concentration-time profiles for morpholine reaction system from simultaneous parameter fitting using Table S1 kinetic parameter estimates, points = experiments, lines = model.*

S9 Linear gradient flow ramps for 2,4-fluoronitrobenzene

To establish the validity of the first order with respect to 2,4-difluoronitrobenzene **1**, linear flow ramp gradients were developed. The relative ratio of the pump flow rates for P1 to P2 were varied to obtain different 2,4-difluoronitrobenzene **1** concentration levels whilst keeping the concentration of morpholine **6** constant (Figure S9). A concentration-time profile was generated for each concentration level of 2,4-difluoronitrobenzene **1** (Figure S10). These experiments confirmed the first order dependency with respect to 2,4-difluoronitrobenzene **1**.

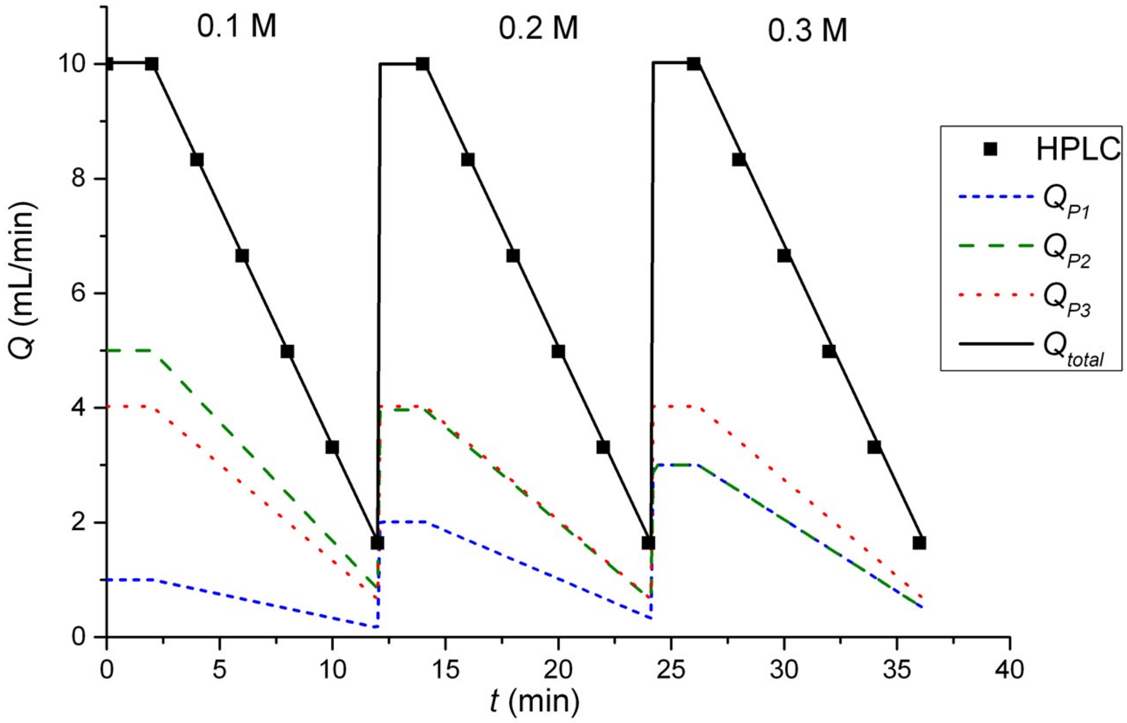

Figure S9. *Changes in volumetric flow rate over time, where Q_{P1}, Q_{P2}, Q_{P3} and Q_{total} were for pump 1 (Ar in EtOH), pump 2 (EtOH), pump 3 (morpholine **6** in EtOH) and total volumetric flow rate respectively. The linear flow ramps keep constant morpholine **6** concentration and vary 2,4-difluoronitrobenzene **1**: (i) 0.1 M, (ii) 0.2 M and (iii) 0.3.*

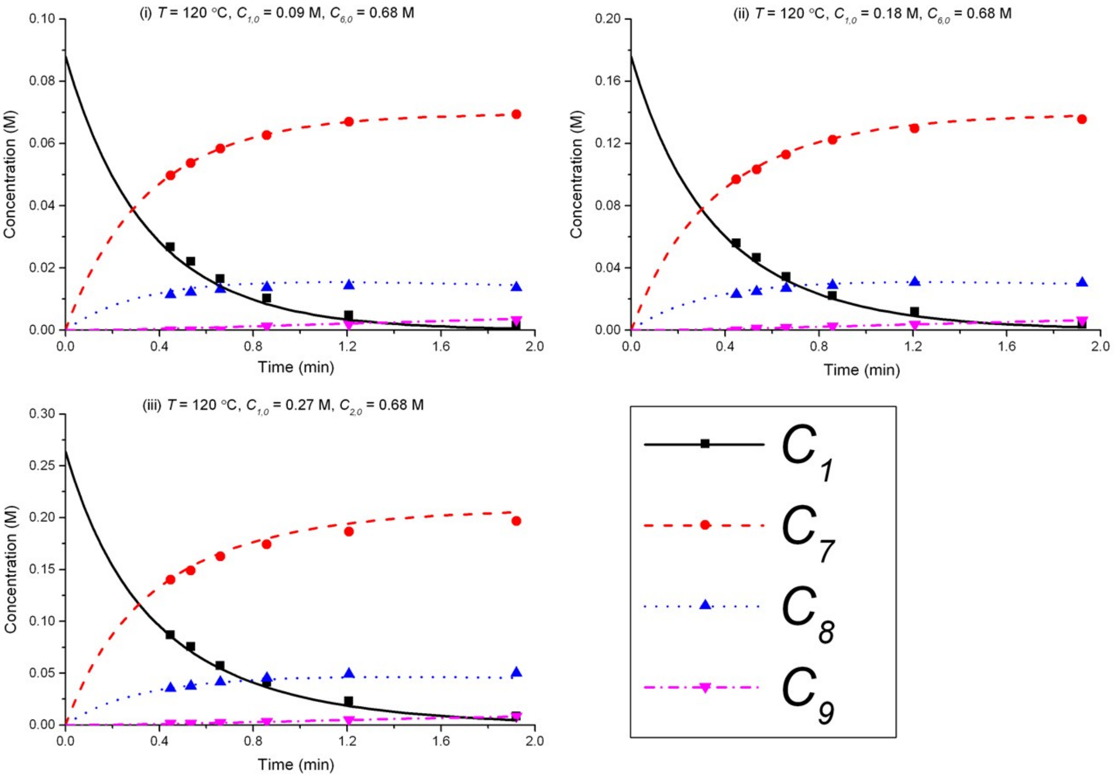

Figure S10. *Concentration-time profiles for T = 120 °C. Points = experiments and lines = model using kinetic parameter estimates in Table S1.*

S10 Comparison to measurements under steady-state conditions

The steady-state experimental data was compared with the flow ramp gradient data. Each residence time point was left for at least three reactor volumes worth of material prior to HPLC injection. The reaction conditions used are shown in the caption in Figure S.9. The data collected at steady-state showed excellent agreement to the kinetic model generated from the unsteady-state flow data (Figure S11).

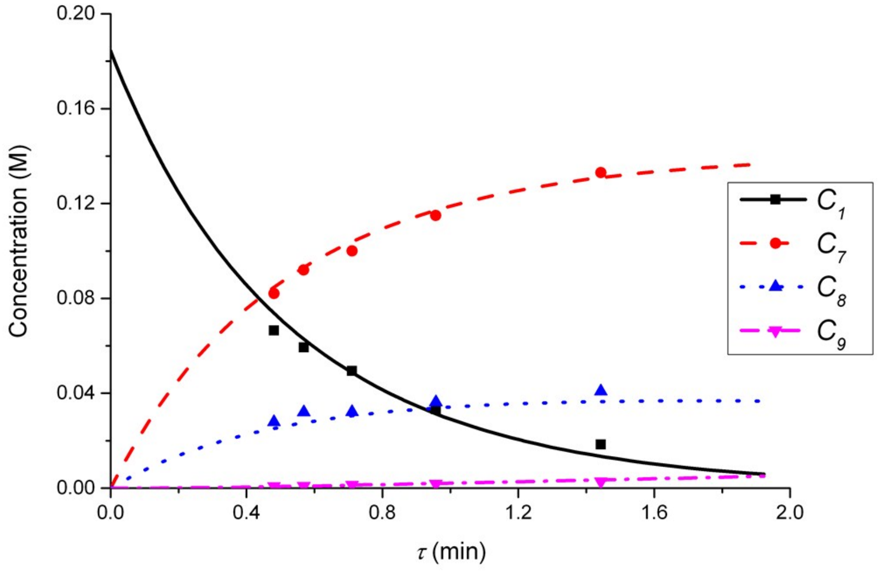

Figure S11. *Unsteady-state kinetic model fit to the steady-state experimental measurements for concentration-time profile at T = 90 °C,* $C_{1,0}$ = 0.18 M $C_{6,0}$ = 1.3 M. *Points = experiments and lines = model using kinetic parameter estimates in Table S1.*

S11 Influence of dispersion on kinetic parameter estimation

In this work we use the residence time in a coiled tube reactor to access kinetics of the S_NAR system studied. If the coil reactor may be described by piston flow (also plug flow) the conversion for a first order reaction is:

$$\frac{C}{C_o} = e^{-k\tau_{res}} \qquad \text{Eq(1)}$$

However, it is well established that dispersion can have a significant effect on the progress of a reaction (see for instance Rosas [1]). In the 1950s Taylor [2] described dispersion in of a solute a straight pipe and this was modified by Aris [3] to give:

$$D_s = D_m + \kappa \frac{d_t^2 u^2}{192\, D_m} \frac{m^2}{s} \qquad \text{Eq(2)}$$

With D_S the dispersion coefficient, D_m the diffusion coefficient, d_t the tube diameter and u the mean velocity in the tube. In liquid systems the term D_m is negligible, and κ represents the ratio of dispersion in a conduit to dispersion in a straight tube with similar diameter; thus for a straight cylindrical tube under laminar flow κ =1. The dispersion ratio κ is dependent on the channel geometry as well as the flow regime (e.g. laminar or turbulent). For laminar flow in coiled tubes the parabolic velocity profile is altered as a result from centrifugal forces. So called Dean vortices form which introduce radial flow, and hence radial mixing which

reduces dispersion. The intensity of the radial flow is characterised by the Dean number ($De = Re\sqrt{\frac{d_t}{d_c}}$ with the Reynolds number $Re = \frac{\rho u d_t}{\mu}$ and d_c the coil diameter). In the 1970s dispersion in coils under laminar conditions was studied experimentally in the group of Vasudeva [4-7] in wide bore tube (4.4–20 mm), and by Van den Berg and Deelder [8] in 0.5 and 1 mm ID tubes. They found that the dispersion ratio κ reduces significantly below 1. Theoretical work by Janssen [9] and Johnson and Kamm [10] showed dispersion ratio in coils may be correlated by $DeSc^{0.5}$. Shetty and Vasudeva [6] fit their data to $\ln(DeSc^{0.5})$ and Iyer and Vasudeva [7] extend the correlation with a second order term to describe data for which Re>100. These, and more recent data from others in small bore tube have been plotted in Figure S12, and it appears that the data is not consistent. We found that the data sets for Re<100 and $d_t \leq 1mm$ could be correlated with

$$\kappa = \left(\frac{(DeSc^{0.5})_{crit}}{De\,Sc^{0.5}}\right)^{\alpha} \qquad \text{Eq(3)}$$
$$with\ \ \alpha = \ln{(DeSc^{0.5})_{crit}}\left(0.068 + 0.087\ln{(DeSc^{0.5})_{crit}}\right)$$

This is similar to the correlation described by Van den Berg and Deelder [8] where $(DeSc^{0.5})_{crit}$ is a critical number below which the coil behaves as a straight tube; it appears this value is unique for each literature data set. For the data measured in wide bore tube by Trivedi and Vasudeva [4] and Iyer and Vasudeva [7] the required value of α is lower than that given by eq(3).

In the 1960s the effect of dispersion was coupled to reaction system. Wehner and Wilhem [11] solved the general equation for the effect of dispersion on the conversion X of a solute due to a first order reaction with rate constant *k*:

$$1 - X = \frac{C}{C_o} = \frac{4\,a e^{\frac{1}{2Pe}}}{(1+a)^2 e^{\frac{a}{2Pe}} - (1-a)^2 e^{-\frac{a}{2Pe}}}\quad with\ a = \sqrt{1 + 4k\tau_{res}Pe} \qquad \text{Eq(4)}$$

In which dispersion is characterised with the dimensionless Péclet number $Pe = \frac{D_s}{uL}$. Eq(3) can be simplified for low values of $k\tau_{res}Pe$ [1] :

[1] This is done by applying a second order Taylor series expansion of $a \approx 1 + 2k\tau_{res}Pe - 2(k\tau_{res}Pe)^2$

$$\frac{C}{C_o} = \frac{4\left(1 + 2k\tau_{res}Pe - 2\left(k\tau_{res}Pe\right)^2\right)e^{\frac{1}{2Pe}}}{(2)^2 e^{\frac{1 + 2k\tau_{res}Pe - 2\left(k\tau_{res}Pe\right)^2}{2Pe}} - \left(2k\tau_{res}Pe - 2\left(k\tau_{res}Pe\right)^2\right)^2 e^{-\frac{1 + 2k\tau_{res}Pe - 2\left(k\tau_{res}Pe\right)^2}{2Pe}}} \qquad \text{Eq(5)}$$

As $k\tau_{res}Pe$ I very small, the second term in the denominator is much smaller and may be neglected so that:

$$\frac{C}{C_o} = \frac{\left(1 + 2k\tau_{res}Pe - 2\left(k\tau_{res}Pe\right)^2\right)e^{\frac{1}{2Pe}}}{e^{\frac{1 + 2k\tau_{res}Pe - 2\left(k\tau_{res}Pe\right)^2}{2Pe}}} \approx e^{\frac{1}{2Pe}\left(-2k\tau_{res}Pe + 2\left(k\tau_{res}Pe\right)^2\right)} \qquad \text{Eq(6)}$$

Here we assumed $2k\tau_{res}Pe \ll 1$ so

$$\frac{C}{C_o} \approx e^{\left(-k\tau_{res} + \left(k\tau_{res}\right)^2 Pe\right)} \qquad \text{Eq(7)}$$

Combining equation (2) and (7) results and observing (i) in liquid systems the term D_m becomes negligible compared to D_s and (ii) $\tau_{res} = \frac{L}{u}$ it follows that:

$$\frac{C}{C_o} \approx e^{-k\tau_{res}\left(1 - k\frac{L}{u}\frac{D_s}{uL}\right)} = e^{-k\tau_{res}\left(1 - k\frac{L}{u}\frac{\kappa\frac{d_t^2u^2}{192\,D_m}}{uL}\right)}$$

$$\frac{C}{C_o} \approx e^{-k\tau_{res}\left(1 - \frac{\kappa}{192}\frac{k\,d_t^2}{D_m}\right)} \qquad \text{Eq(8)}$$

This shows that in a coil the observed rate constant k_{obs} may be given as:

$$k_{obs} = k\left(1 - \frac{\kappa}{192}\frac{kd_t^2}{D_m}\right) = k\left(1 - \frac{\kappa}{192}Da_r\right) \qquad \text{Eq(9)}$$

Where the Damköhler number for radial diffusion may be defined as $Da_r = \frac{kd_t^2}{D_m}$. The relative error ϵ_{D_C} in the observed rate constant k_{obs} obtained from the profiles measured in continuous flow in a coil with respect to the true rate constant k will be

$$\epsilon_{D_C} = \frac{k_{obs} - k}{k} = - \frac{\kappa}{192} Da_r \times 100\% = - \frac{k d_t^2}{1.92 D_r} \left(\frac{(DeSc^{0.5})_{crit}}{De\, Sc^{0.5}} \right)^{\alpha} \% \qquad \text{Eq(10)}$$

In our case we measured an F-curve at 6 min residence time, obtained a dispersion ratio of $\kappa = 0.31$ at $DeSc^{0.5} = 65$ (this uses a typical value for $D_m = 0.8\ 10^{-9}\ m^2/s$ in ethanol [12]). This corresponds well with the data from Van den Berg and Deelder [8]. Using equation 13 with $(DeSc^{0.5})_{crit} = 11$ and α = 0.66 we plotted the range of κ, 1 to 0.7 for our experiments conditions, in Figure S12. With $\frac{d_t^2}{D_m} = 788$ and κ = 1 (worst case scenario) the error k is $\epsilon_{D_C} \approx 4.1k$. For a second order reaction the reaction at a particular excess of pyrrolidine the rate constant k may be approached by $k \approx k_2 C_{pyrr.o}$. Table S3 shows ϵ_{D_C} corresponding to the largest rate constant in Table 1. For the profiles where the reactant has fully converted or no stating material is available at all residence times no sensible estimate dispersion effect on k can be made and these conditions are indicated by shading the cells in table S3. The other rates are a factor 10 lower, and this will result in significantly lower errors, Table S4, thus the error in the rate constants with dispersion is very low for the overreaction pathways.

In conclusion, the estimated rate parameters may be up to 10% higher than those in Table 1, for the fastest rate up to 20%. This is based on a worst case scenario of a dispersion ratio of 1.

Table S3. Estimated error in the rate constants predicted from coils with dispersion for step 1 and step 2 (maximum disappearance of starting material). C/C_o is >11% in the shaded cells, and ≤11% in the other cells.

		Temperature (°C)			
		30	60	90	120
Molar Excess pyrrolidine	1.5	1%	4%	11%	26%
	4	3%	11%	30%	69%
	7	6%	19%	52%	121%

Table S4: Estimated error in the rate constants predicted from coils with dispersion for step 3 and step 4. C/C_o is ≤5% for all experiments.

		Temperature (°C)			
		30	60	90	120
Mo lar	1.5	0%	0%	0%	1%

Excess pyrrolidine	4	0%	0%	1%	3%
	7	0%	1%	2%	5%

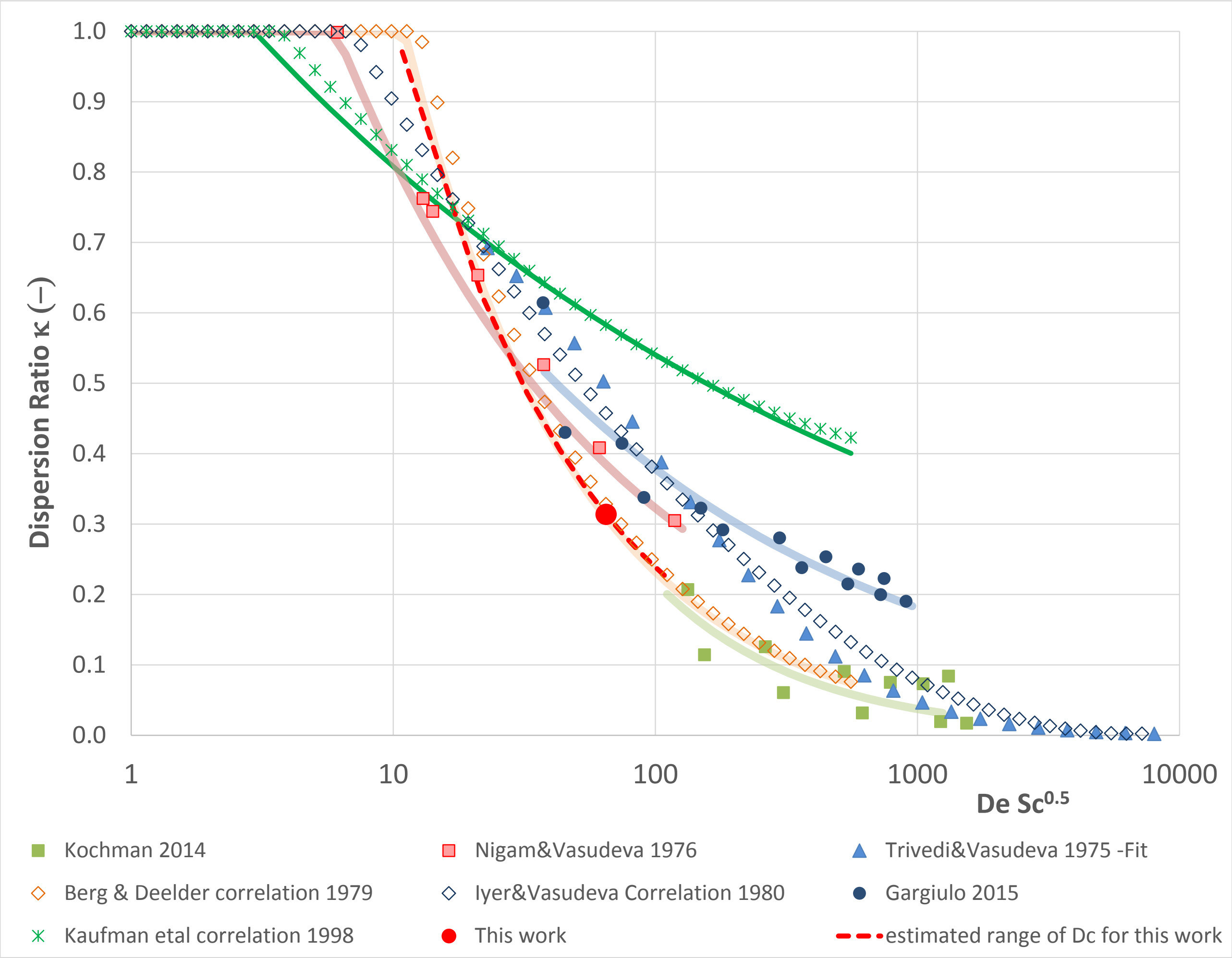

Figure S12. The dispersion ratio κ vs DeSc$^{0.5}$ for various literature data. Where possible data have or correlations have been taken from the papers. If the data could not be retrieved and no correlation was given the data has been fitted with a line that visually represents the data well.

S12 Dispersion experiment

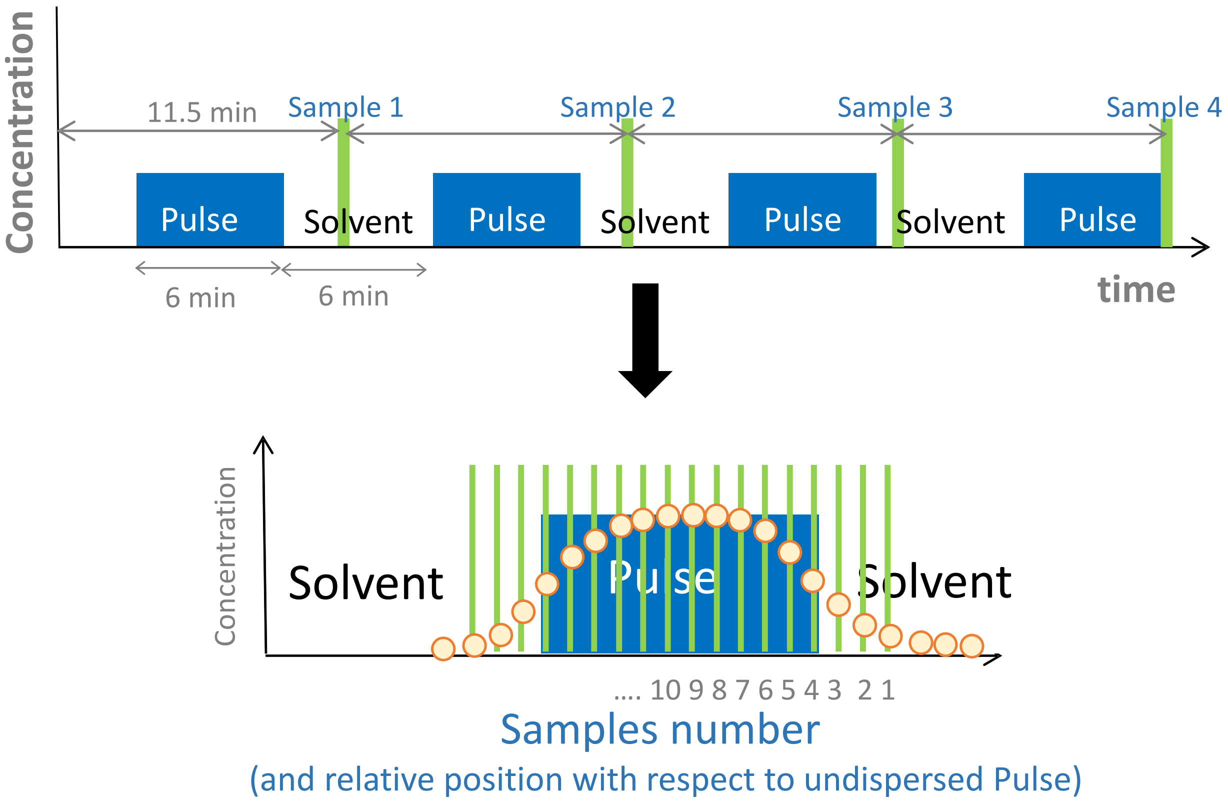

Figure S13. Alternate periods of biphenyl pulse and solvent at 6 min intervals, with online samples acquired at 10.5 minute intervals. The samples were acquired at different points of the pulses, thus recreating multiple samples of a single pulse.

Two pump reservoir solutions were prepared with biphenyl (Sigma Aldrich 99.9%) solution in ethanol (9.75 mmol L^{-1}) (VWR 99.96%) and acetone (VWR 99.9%). The pump feeds were mixed using a Swagelok tee-piece and fed through a PTFE coil of 6.1 mL (0.79 mm internal diameter, length 1232 cm, volume 6.1 mL from the tee-piece to the sample loop) before online HPLC analysis using a 4-port microvolume sampling loop (0.06 μL volume). The pumps were calibrated before running the experiment. The volume was measured by recording the time it taken for a dye tracer segmented by an air bubble to flow from the tee-piece to the sample loop at 1 mL min^{-1}.The solvent pump was set to pump at 1.017 mL min^{-1} for 2.5 min followed by alternate periods of the biphenyl and solvent pump at 1.017 mL min^{-1} for 6 min intervals. The sample loop was set to acquire at 11.5 minute intervals, thus obtaining samples at different points through the pulse giving a representation of multiple samples at 30 s intervals of a single pulse (Figure S13).

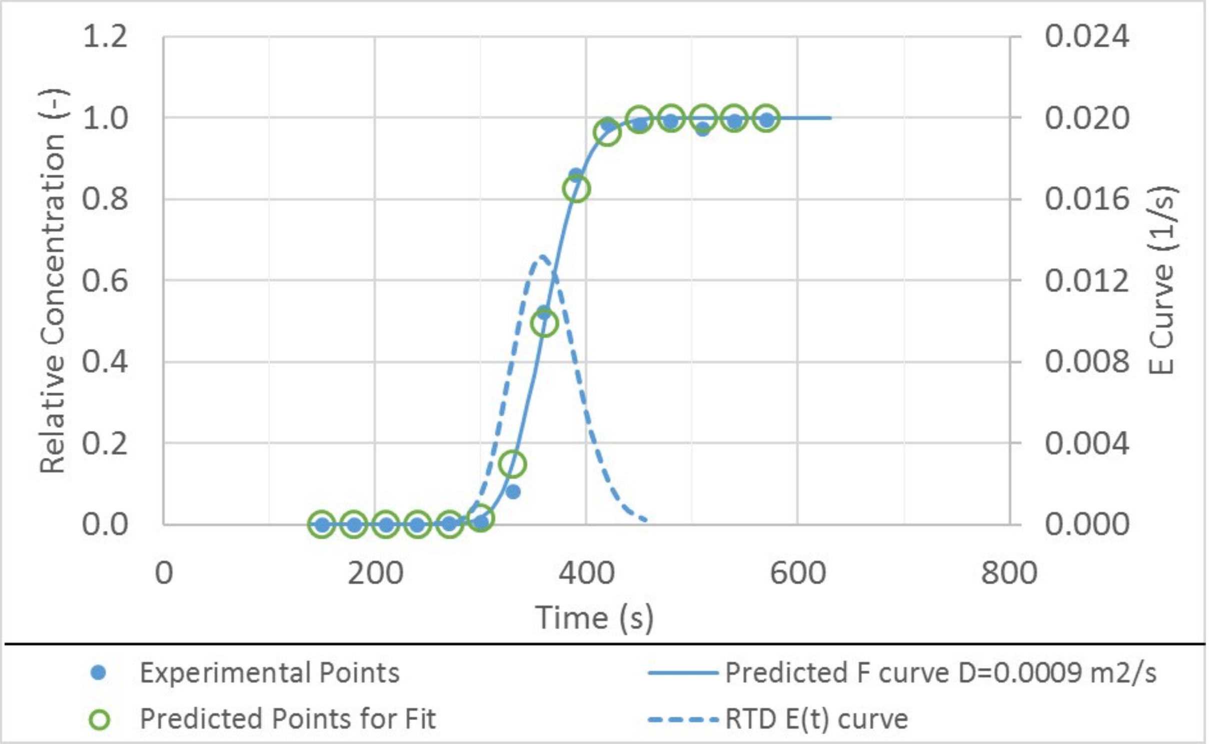

Figure S14. Predicted F curve and RTD E(t) curve using the experimental data.

S13 References

1. C. B. Rosas, *Industrial and Engineering Chemistry Fundamentals*, 1969, **8**, 361-364.
2. G. Taylor, *Proceedings of the Royal Society of London A: Mathematical, Physical and Engineering Sciences*, 1953, **219**, 186-203.
3. R. Aris, *Proceedings of the Royal Society of London A: Mathematical, Physical and Engineering Sciences*, 1956, **235**, 67-77.
4. R. N. Trivedi and K. Vasudeva, *Chem Eng Sci*, 1975, **30**, 317-325.
5. K. D. P. Nigam and K. Vasudeva, *Chem Eng Sci*, 1976, **31**, 835-837.
6. V. D. Shetty and K. Vasudeva, *Chem Eng Sci*, 1977, **32**, 782-783.
7. R. N. Iyer, *Chem Eng Sci*, 1981, **36**, 1104-1105.
8. J. H. van den Berg and R. S. Deelder, *Chem Eng Sci*, 1979, **34**, 1345-1347.
9. L. A. M. Janssen, *Chem Eng Sci*, 1976, **31**, 215-218.
10. M. Johnson and R. D. Kamm, *Journal of Fluid Mechanics*, 1986, **172**, 329-345.
11. J. F. Wehner and R. H. Wilhelm, *Chem Eng Sci*, 1956, **6**, 89-93.
12. E. E. Hills, M. H. Abraham, A. Hersey and C. D. Bevan, *Fluid Phase Equilibr*, 2011, **303**, 45-55.
